# New binary and ternary SiO_2_ composites with Fe_2_O_3_ and Co_2.74_O_4_ and the evaluation of their γ-radiation shielding properties

**DOI:** 10.1038/s41598-025-93991-6

**Published:** 2025-04-21

**Authors:** Ahmed M. A. El-Seidy, Mohamed S. El-Okaily, Islam M. Nabil, Amany A. Mostafa

**Affiliations:** 1 Inorganic Chemistry Department, Advanced Materials Technology and Mineral Resources Research Institute, National Research Centre, El-bohouth St., P.O. 12622 Dokki, Cairo Egypt; 2 Refractories, Ceramics and Building Materials Department (Biomaterials Group), Advanced Materials Technology and Mineral Resources Research Institute, National Research Centre, Cairo, Egypt; 3 Physics Department, Faculty of Science, Fayoum University, Fayoum, Egypt

**Keywords:** Binary nanocomposites, Ternary nanocomposites, Mesoporous SBA-15, $\gamma$-radiation shielding, Chemistry, Inorganic chemistry, Materials chemistry, Chemical synthesis

## Abstract

A new series of binary and ternary nanocomposites contains cobalt oxide or iron/cobalt oxides were manufactured to increase silicon dioxide $\gamma$ shielding power. XRD indicated the presence of Co as Co_2.74_O_4_ (COD: 1528446) and the presence of iron as Fe_3_O_4_ (COD: 9002318 and 9005814). Using Profex, the Rietveld refinements were carried out. The R_w_, R_ex_, x^2^, and G_of_ were 4.49, 4.34, 1.07, and 1.03, respectively, indicating good refinement parameters. XPS indicated the presence of Si ($\hbox {Si}^{4+}$), Fe (Fe$_{2}$O$_{3}$) and cobalt ($\hbox {Co}^{2+}$ and $\hbox {Co}^{3+}$). TEM analysis showed that all metal oxide@SBA-15 solids have characteristic and well-organized SBA-15 structures. The $\gamma$-radiation shielding for the prepared samples were investigated via the Monte-Carlo code (MCN) and Phy-X software. The results confirmed that, adding high concentrations of cobalt-oxide and hematite increases the linear attenuation significantly. The SiCoFe-3 sample, which contains the highest content of cobalt-oxide and hematite, has the best $\gamma$-radiation shielding capability among all the synthesized SiCo/SiCoFe samples.

## Introduction

Nuclear energy is considered one of the most widespread energy sources in many diverse countries worldwide due to its high efficiency, low cost, and low carbon emissions, solving global climate change problems^1^. The increasing reliance on nuclear energy as a sustainable, low-carbon power source underscores the urgent need for advanced radiation shielding materials to mitigate the risks posed by $\gamma$-rays and neutrons in nuclear installations and radioactive waste management systems^2^. While nuclear energy addresses global climate challenges through high efficiency and minimal greenhouse emissions, its inherent hazards-particularly ionizing radiation-demand robust shielding solutions to protect both human health and the environment^3^. Traditional materials such as lead, polymer composites^4^, clay composites, concrete composites, and different glasses^4,5^ have been widely employed but face limitations, including toxicity, bulkiness, and reduced effectiveness at high radiation doses. Recent advancements in nanotechnology have catalyzed the development of novel shielding materials that combine high atomic number (Z) elements with nanostructured matrices to enhance radiation attenuation while maintaining mechanical and thermal stability^4^

Nanomaterials, particularly those incorporating heavy metal oxides (HMOs), offer superior shielding performance due to their high surface-to-volume ratio, uniform dispersion of active elements, and tunable structural properties. For instance, polymer composites embedded with nano-sized HMOs demonstrate enhanced $\gamma$-ray attenuation by maximizing photon interactions via the photoelectric effect, Compton scattering, and pair production^6,7^ . Similarly, silica-based nanomaterials, such as mesoporous silica SBA-15, have emerged as promising matrices due to their high surface to volume ratio, chemical stability, and customizable pore architecture^7,8^ . Silica nanoparticles have extensive applications in several fields, such as catalysis^9^, pigments^10^, pharmaceuticals^11^, electronics^12^, insulators^13^and facilitate the integration of functional HMOs. Recent studies highlight the efficacy of silica nanocomposites doped with transition metal oxides (e.g., Fe$_{3}$O$_{4}$ and Co$_{3}$O$_{4}$) in achieving synergistic shielding effects, where the high-Z metal oxides amplify radiation absorption while the silica framework ensures structural integrity and thermal resistance^14^.

Researchers have given significant attention to them because of their large specific surface area (>1000 $\hbox {m}^{2}$ $\hbox {g}^{-1}$), adjustable pore size, limited pore diameter dispersion, and exceptional radiochemical and structural durability^15,16^. In separation and decontamination procedures, MSNs, sometimes known as popular ligand-solid supports, are often used to remove radionuclides selectively^17,18^. Moreover, their non-swelling characteristic, good chemical stability, and decreased functional group steric hindrance make them valuable for applications involving radioactive waste disposal^18^

Cobalt-based nanomaterials, notably cobalt ferrites (CoFe$_{3}$O$_{4}$) and cobalt oxides (Co$_{3}$O$_{4}$), are particularly advantageous for $\gamma$-ray shielding due to their high density, magnetic properties^19^, and exceptional radiation stability^14^. Alhindawy et al. (2023) demonstrated that Co-TiO$_{2}$ hybrid nanomaterials significantly improve shielding efficiency, attributing this to cobalt’s high electron density and capacity to disrupt photon energy through multiple scattering mechanisms^14^. Similarly, iron oxides (Fe$_{3}$O$_{4}$) contribute to neutron moderation and structural reinforcement, as evidenced by Mahmoud et al. (2024)^20^, who reported enhanced radiation attenuation in concrete composites modified with nano-Fe$_{3}$O$_{4}$. Despite these advancements, the integration of cobalt and iron oxides within a mesoporous silica framework remains underexplored, particularly in optimizing their synergistic effects for multifunctional shielding applications.

This study addresses this gap by designing a two-dimensional (2D) nanostructured composite of cobalt and iron oxides embedded in SBA-15 mesoporous silica. The rationale for this approach lies in leveraging the unique properties of SBA-15-such as its ordered pore structure and high thermal stability-to host uniformly dispersed Co$_{3}$O$_{4}$ and Fe$_{2}$O$_{3}$ nanoparticles. The incorporation of cobalt enhances photon absorption through its high-Z nuclei, while iron oxides improve mechanical durability and neutron moderation. Furthermore, the mesoporous architecture of SBA-15 maximizes the interfacial interactions between radiation and the composite, thereby enhancing attenuation efficiency. Recent work by Taghavimoghaddam et al. (2012)^21^ . underscores the potential of SBA-15 as a scaffold for metal oxide catalysts, yet its application in radiation shielding remains nascent. By systematically evaluating the $\gamma$-ray shielding performance, thermal stability, and structural coherence of this novel composite, this research aims to establish a paradigm for next-generation shielding materials that balance efficacy, sustainability, and practical deployability.

The significance of this work extends beyond nuclear safety; it aligns with global efforts to develop eco-friendly, high-performance materials for applications in aerospace, medical radiation therapy, and radioactive waste containment. By integrating cutting-edge nanotechnology with established principles of radiation physics, this study contributes to the evolving discourse on material science-driven solutions for a safer and sustainable nuclear future.

## Materials and methods

There was no purifying procedure before the chemicals were administered. The oxides and elemental fraction weight and density of the synthetic SiCoFe glasses are illustrated in Table 1.

**Table 1:** The elemental fractional abundance and density of the prepared SiCoFe glasses.

Samples ID	Oxides composition (mol%)	Elemental composition (mol%)				Density (g$\cdot$cm^-3^)
		Si	O	Co	Fe	
SiCo-1	70.2% SiO_2_ + 29.8 Co_2.74_O_4_	32.82	40.95	26.23	–	1.56
SiCo-2	16.8% SiO_2_ + 83.2% Co_2.74_O_4_	07.84	18.88	73.27	–	2.36
SiCoFe-1	52.6% SiO_2_ + 22.5 Co_2.74_O_4_ + 24.9% Fe_2_O_3_	05.26	38.19	19.79	17.42	1.7
SiCoFe-2	14.5% SiO_2_ + 71.7% Co_2.74_O_4_ + 13.8% Fe_2_O_3_	06.79	20.45	63.11	09.65	2.38
SiCoFe-3	12.5% SiO_2_ + 63.6% Co_2.74_O_4_ + 23.9% Fe_2_O_3_	05.85	21.45	55.95	16.74	2.41

### Preparation of SBA-15

A solution of 2 g (0.35 mmol) of Pluronic (P123, Sigma-Aldrich) in 90 mL of 2M HCl (37%, Sigma-Aldrich) plus 22.50 mL of distilled water was stirred to prepare the mesoporous silica SBA-15. Once the solution was fully dissolved, 4.25 g (20.4 mmol) of tetraethyl orthosilicate (Sigma-Aldrich) was added to the mixture dropwise while stirring continuously at room temperature. After a 24-h static ageing period at 100 $^{\circ }$C, the mixture was filtered, washed with distilled water, and dried at 90 $^{\circ }$C for 30 minutes. The powder was heated to 550 $^{\circ }$C for 6 h at a rate of 2 $^{\circ }$C/minute during the calcination process.

### Synthesis of nano-composites

Table 2 shows the results of preparing nanocomposites with varying percentages of iron, cobalt, and silicon oxide using the SBA-15 that had been previously prepared. To carry out the impregnation, a 150 mL solution of cobalt hydroxide and a 100 mL solution of iron hydroxide were added to an SBA-15 dispersion while stirring for 2 h. Subsequently, the solution was added to a bath that was maintained at 90 $^{\circ }$C with constant stirring until it evaporated completely. We calcined the powders that came out of it at 550 $^{\circ }$C for 6 h, increasing the temperature by 2 $^{\circ }$C per minute. The mechanism for synthesizing a cobalt oxide-iron oxide impregnated SBA-15 may involve the impregnation process, involving pore filling with cobalt salt and/or iron salt solutions followed by the transformation of these salts into oxides through thermal decomposition^22^.

**Table 2:** Starting materials.

Samples ID	SiO_2_		Co(NO_3_)_2_$\cdot$3H_2_O		Fe(NO_3_)_3_$\cdot$9H_2_O	
	mass (g)	mmole	mass (g)	mmole	mass (g)	mmole
SiCo-1	3.00	49.96	7.41	25.45	–	–
SiCo-2	0.50	8.32	14.81	50.90	–	–
SiCoFe-1	3.00	49.93	7.41	25.45	10.87	26.91
SiCoFe-2	1.50	24.96	44.45	152.72	10.85	26.86
SiCoFe-3	0.50	8.32	14.82	50.91	7.23	17.91

### Theoretical basis $\gamma$-rays

### Attenuation

The linear attenuation (LA) (using Lambert–Beer’s law) can be used to identify shielding materials^23^:

1 $$\begin{aligned} MA=\frac{LA}{\rho }, \end{aligned}$$

I and Io are the intensity of transferred $\gamma$-rays through the material and in air, respectively, x is the thickness of the attenuator, and LA is the L-a coefficient. The following is the formula for calculating the mass attenuation coefficient (MA), a crucial indicator of a material’s property^24–26^:

2 $$\begin{aligned} LA=-\frac{1}{x}ln\left( \frac{I}{I_{o}}\right) , \end{aligned}$$

The LA parameter calculates the values needed to reduce the incident radiation to half or a tenth of its original value. The half-value layer (HV) and the tenth-value layer (TV) can be calculated using Eqs. (3) and (4)^20,27^:

3 $$\begin{aligned} HV=\frac{ln\hspace{2pt} 2}{LA}, & \end{aligned}$$

4 $$\begin{aligned} TV=\frac{ln10}{LA}, & \end{aligned}$$

The mean-free path (MF) describes the distance that $\gamma$-ray photons travel inside the shielding material between two successive collisions, which is determined through Eq. (5)^20,27^:

5 $$\begin{aligned} MF=\frac{1}{LA}, & \end{aligned}$$

## Methods

### Characterization

Cobalt, iron, and silicon oxidation states were ascertained using XPS. The K-$\alpha$ instrument, manufactured by Thermo Fisher Scientific in the USA, was used to gather XPS images. The instrument employed monochromatic X-ray Al K$\alpha$ radiation with a spot size of 400 $\upmu$m and a pressure of $10^{-9}$ mbar. The full spectrum pass energy was 200 eV, and the narrow spectrum was 50 eV. The use of X-ray diffraction allowed for the investigation of the glasses’ structural properties as well as their crystallite size. In the range of 2$\theta$ = $5^{\circ }$ - $60^{\circ }$, the XRD patterns were acquired using a Cu-radiation ($\lambda$ = 1.542Å) at 45 K.V. and 35mA. The average crystallite sizes were determined using the Debye–Scherrer Equation (6).

6 $$\begin{aligned} D = K \lambda / \beta Cos \theta \end{aligned}$$

Where D is the nano-particles crystalline size, K represents the Scherrer constant (0.98), $\lambda$ denotes the wavelength (1.54), $\beta$ denotes the full width at half maximum (FWHM). A SEM of the type QUANTA FEG 250, manufactured by EDX Philips in the USA, was used to study the morphology. This powder was analyzed using a transmission electron microscope to determine its size, shape, and surface structure. The JEOL 2100 LB6 transmission electron microscope was used for the TEM analysis. Nanomotors were prepared and analyzed using Fourier transform infrared spectroscopy to learn about molecular vibration and atomic bonding. In the 4000–400 $\hbox {cm}^{-1}$ range, with a 2 $\hbox {cm}^{-1}$ spectral resolution and a 2 $\hbox {mm s}^{-1}$ scan speed, the KBr disk technique was applied using a Vertex 70 spectrometer (Bruker Optiks, Germany). Gas absorption in accordance with Brunauer-Emmet-Teller (BET) method 41 forms the basis of the surface area measurement techniques. The BET surface area (SBET) was determined through N$_{2}$ sorption analysis, and the pore size distribution (PSD) was assessed using non-local density functional theory (NL-DFT) for cylindrical pores. The BELSORP MAX, a pore size and surface area analyzer made in Japan, was used for the N$_{2}$ physisorption experiments. The samples underwent a 3-h outguessing process in a high vacuum at 350 $^{\circ }$C prior to analysis. After allowing 4 minutes for equilibration between each point, the adsorption/adsorption isotherms were obtained at -196 $^{\circ }$C. The BELMaster Data Analysis software was used to determine the textural properties from the isotherms. With p/p0 = 0.97, the pore volume was assessed using the isotherm’s adsorption branch.

### MCNP simulation

**Fig. 1:**
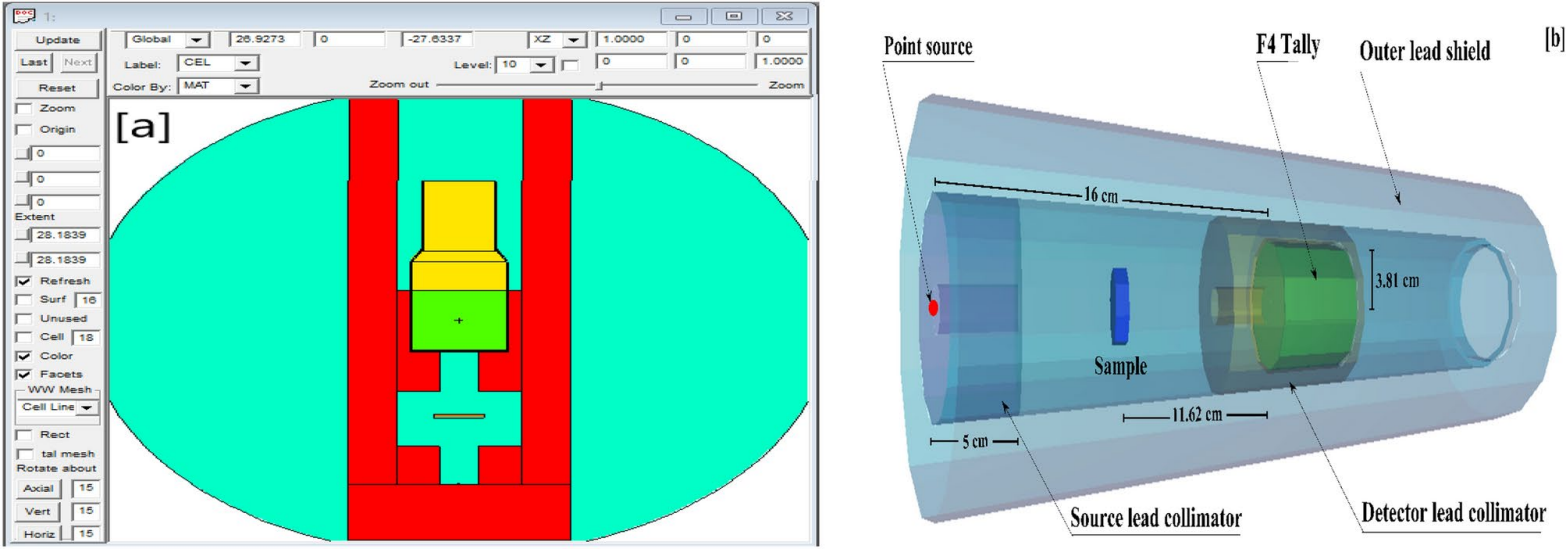
The created radiation shielding simulation system in (**a**) 2D and (**b**) 3D views for the synthetic SiCoFe samples.

The $\gamma$-radiation (G-R) simulations of the synthetic glasses were achieved using MCN with a single energy point source in the E$\gamma$ (0.015–15 MeV). It stimulates the crossing of X-rays and gamma rays while taking into account the ethics of physical interactions including photoelectric (Pee), Compton scattering (COM), and pair production processes (PaP)^28^. The input files of the MCN code need detailed data using a cell card in a text file described the $\gamma$-source, sample, primary and secondary collimator, and detector^29,30^. Figure 1a,b represents the dimensions of the G-R simulated system used for the synthetic SiCoFe glasses radiation shielding investigation. The glasses were created as cylinders and located between the source and the detection cell. The elemental composition of the synthetic SiCoFe samples was created in the material card of the input file. The NPS card was 10000000/run which used for all computations to ensure that the random statistical errors do not exceed 1% .

### Phy-X/PSD software

Online Phy-X software (PhyX) calculates several shielding and attenuation factors for the compositions of materials, dosimetry, etc.^31^. To examine the samples under study, all of the radiation shielding parameters were chosen as output data^32^. The relative differences ($\Omega$ $\%$) were also calculated by comparing the data obtained from PhyX and MCN as follows^33^:

7 $$\begin{aligned} \Omega (\%) = \left| \frac{MCN-PhyX}{MCN} \right| \times 100. \end{aligned}$$

## Results and discussion

### XPS

**Fig. 2:**
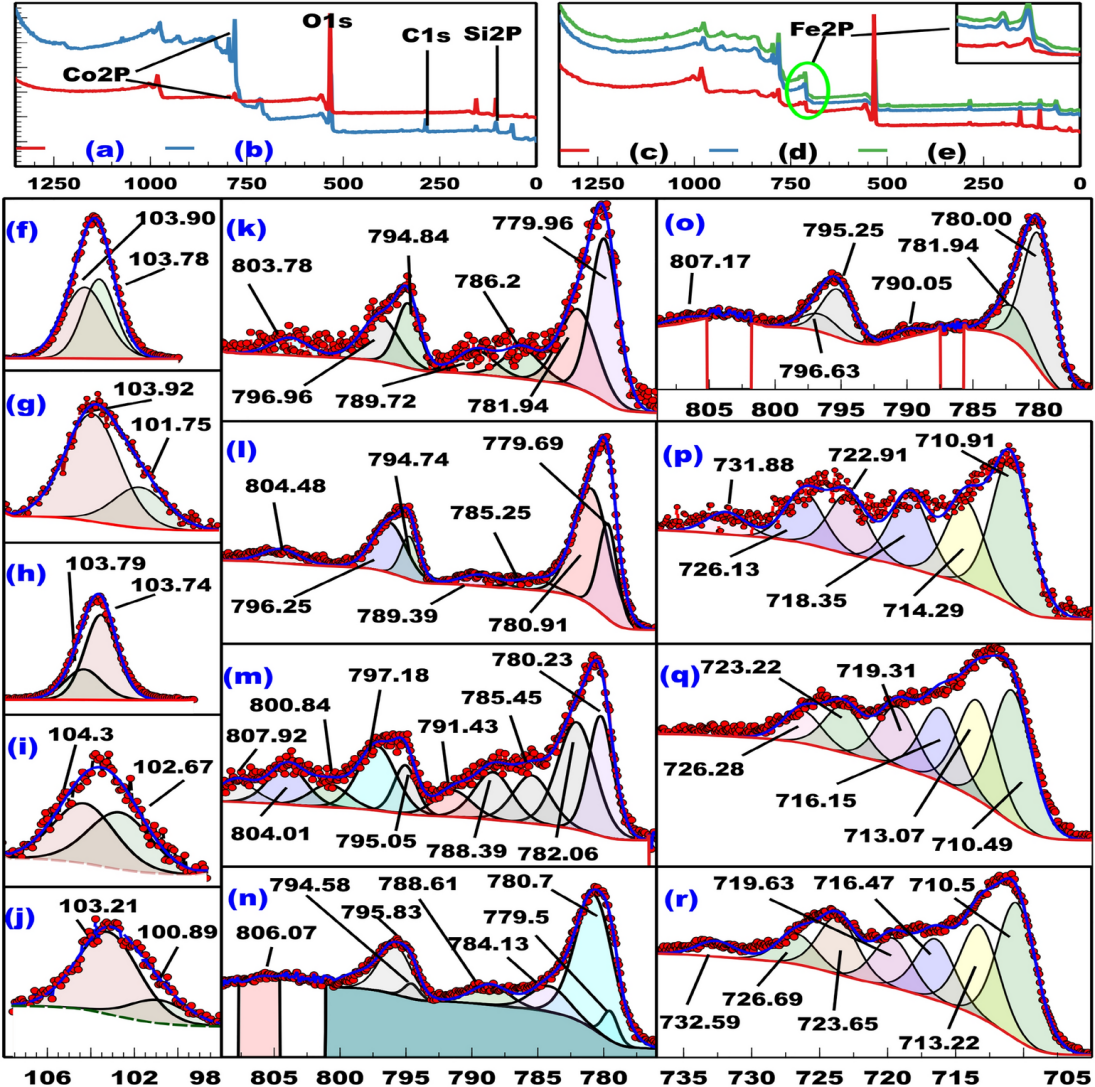
The XPS survey spectra of (**a**) SiCo-1, (**b**) SiCo-2, (**c**) SiCoFe-1, (**d**) SiCoFe-2, and (**e**) SiCoFe-3, The H-R-s Si-2p XPS (**f**) SiCo-1, (**g**) SiCo-2, (**h**) SiCoFe-1, (**i**) SiCoFe-2, and (**j**) SiCoFe-3, The H-R-s Co-2p XPS (**k**) SiCo-1, (**l**) SiCo-2, (**m**) SiCoFe-1, (**n**) SiCoFe-2, and (**o**) SiCoFe-3, and The H-R-s Fe-2p XPS (**p**) SiCoFe-1, (**q**) SiCoFe-2, and (**r**) SiCoFe-3.

The XPS survey spectra of SiCo-1, SiCo-2, SiCoFe-1, SiCoFe-2, and SiCoFe-3 are shown in Fig. 2a–e. They indicate the purity of the nanomaterials showing Si-2p and Co-2p in all spectra indicating the presence of silicon and cobalt while only that of SiCoFe-1, SiCoFe-2, and SiCoFe-3 showed Fe-2p which indicate the presence of iron. The presence of C-1s in all spectra may be due to the adsorption on the surface of the sample or as a residue as discriped in litrature^34–36^. The fitted curves came with good agreement with the experimental curves in all high-resolution (H-R-s) spectra. The H-R-s O-1s XPS spectra show peak in the 529.67–529.98 eV region in all spectra and at 530.95 eV in the spectrum of SiCoFe-1, which may be attributed to M-O and surface adsorbed oxygen (O$_{\beta }$), respectively^37^. Peaks appearing around 533 eV in the spectra of SiCo-1 and SiCoFe-1 may be due to adsorbed water oxygen while that appearing at 531.30 eV in the spectra of SiCo-2 and at 531.07 eV in the spectra of SiCoFe-2 are attributed to surface -OH group^38–40^. The spectra of SiCo-1 and SiCoFe-1 show peaks in the 532.87–532.89 eV region may be attributed to chemisorbed oxygen, while that of SiCoFe-2 and SiCoFe-3 show peaks in the 532.21–532.49 eV region corresponding to superoxide species resulting from the reaction of molecular oxygen and oxygen vacancies^41^. The H-R-s Si-2p XPS spectra are shown in Fig. 2f–j. The spectrum of SiCo-2 shows a peak at 101.75 eV which can be attributed to Si-O, while that of SiCoFe-3 shows a peak at 100.89 eV which can be attributed to silicon core with surface oxidation, respectively^42,43^. The spectrum of SiCoFe-3 shows a peak at 103.21 eV which is attributed to $\hbox {Si}^{4+}$^44^. The spectra of SiCo-1 and SiCo-2 show peaks in the 103.90–103.92 eV region, which can be attributed to SiO_x_ while that of SiCo-1 and SiCoFe-1 show a peak in 103.78–109.79 eV region which can be attributed to SiO_2_^42,45^. The spectra of SiCoFe-2 Show a peaks at 104.30 and 102.67 eV which can be attributed to SiO$_{2}$ (quartz) and Si-O, respectively.^46,47^. The H-R-s Co-2p XPS spectra are shown in Fig. 2k–o. The fitting result of Co-2p show characteristic peaks for Co^2+^ and Co^3+^, confirming the formation of Co_3_O_4_^48^. The spectra of SiCo-1, SiCo-2, and SiCoFe-2 show peaks in the 779.50–779.96, 794.58–794.84, 780.70–781.94, and 795.83–796.96 eV regions, which can be attributed to Co^3+^-2p$_{3/2}$, Co^3+^-2p$_{1/2}$, Co^2+^-2p$_{3/2}$, and Co^2+^-2p$_{1/2}$, respectively^49,50^. These spin-orbit peaks appear in the 780.00–780.23, 795.05–795.25, 781.94–782.06, and 796.63–795.83 eV regions, respectively in the spectra of SiCoFe-1, and SiCoFe-3^42,51^. The Co^2+^ shake-up satellite peaks were shown around 784 (SiCoFe-1), 785 (SiCo-2, and SiCoFe-1), 786 and 803 (SiCo-1), 804 (SiCoFe-1, and SiCoFe-2) while a Co^3+^ shake-up satellite peaks were shown around 789 (SiCo-1, and SiCo-2), 790 (SiCoFe-3), 806 (SiCoFe-2) and 807 eV (SiCoFe-1, and SiCoFe-3)^52–54^. The ratio of Co^3+^ can be calculated from Eq. (8) and found to be 56.00%, 30.36%, 38.59%, 9.19%, and 74.70% in SiCo-1, SiCo-2, SiCoFe-1, SiCoFe-2, and SiCoFe-3, respectively.

8 $$\begin{aligned} \% [Co^{3+}] = \frac{(\sum peak \hspace{2pt} erea \hspace{2pt} Co^{3+} \hspace{2pt} (2p_{3/2} + 2p_{1/2})) * 100}{\sum Peak \hspace{2pt} erea \hspace{2pt} Co^{3+} \hspace{2pt} (2p_{3/2} + 2p_{1/2}) \hspace{2pt} + \hspace{2pt} Co^{2+} \hspace{2pt} (2p_{3/2} + 2p_{1/2})} & \end{aligned}$$

The H-R-s Fe-2p XPS spectra of SiCoFe-1 (Fig. 2p), SiCoFe-2 (Fig. 2q) and SiCoFe-3 (Fig. 2r) showed two spin-orbit doublets (2p_3/2_ and 2p_1/2_) at binding energies (BE) in 710.49–710.91 eV and 722.91–723.65 eV regions, respectively, which are consistent with Fe^+3^ in Fe_2_O_3_ with the electronic configuration 3d^5^^55,56^. As expected, Fe-2p_3/2_ with degeneracy of four multiples is stronger and narrower than Fe-2p_1/2_ with only two j-j coupling^57^. The spectra of SiCoFe-2 and SiCoFe-3 show peaks in the 713.07–713.22 eV regions resulting from 3d $\rightarrow$ 4s electronic transition due to an electron ejection from Fe-2p^58^. All spectra show peaks in the 714.29–719.63 eV region which are characteristic of Fe(III) species^59,60^. The peak around 726 eV in all spectra indicate a different environment surrounding part of Fe^3+^^39^. Peak around 731 and 732 eV in the spectra of SiCoFe-1 and SiCoFe-3, respectively, are satellite peaks^61^.

### XRD

**Fig. 3:**
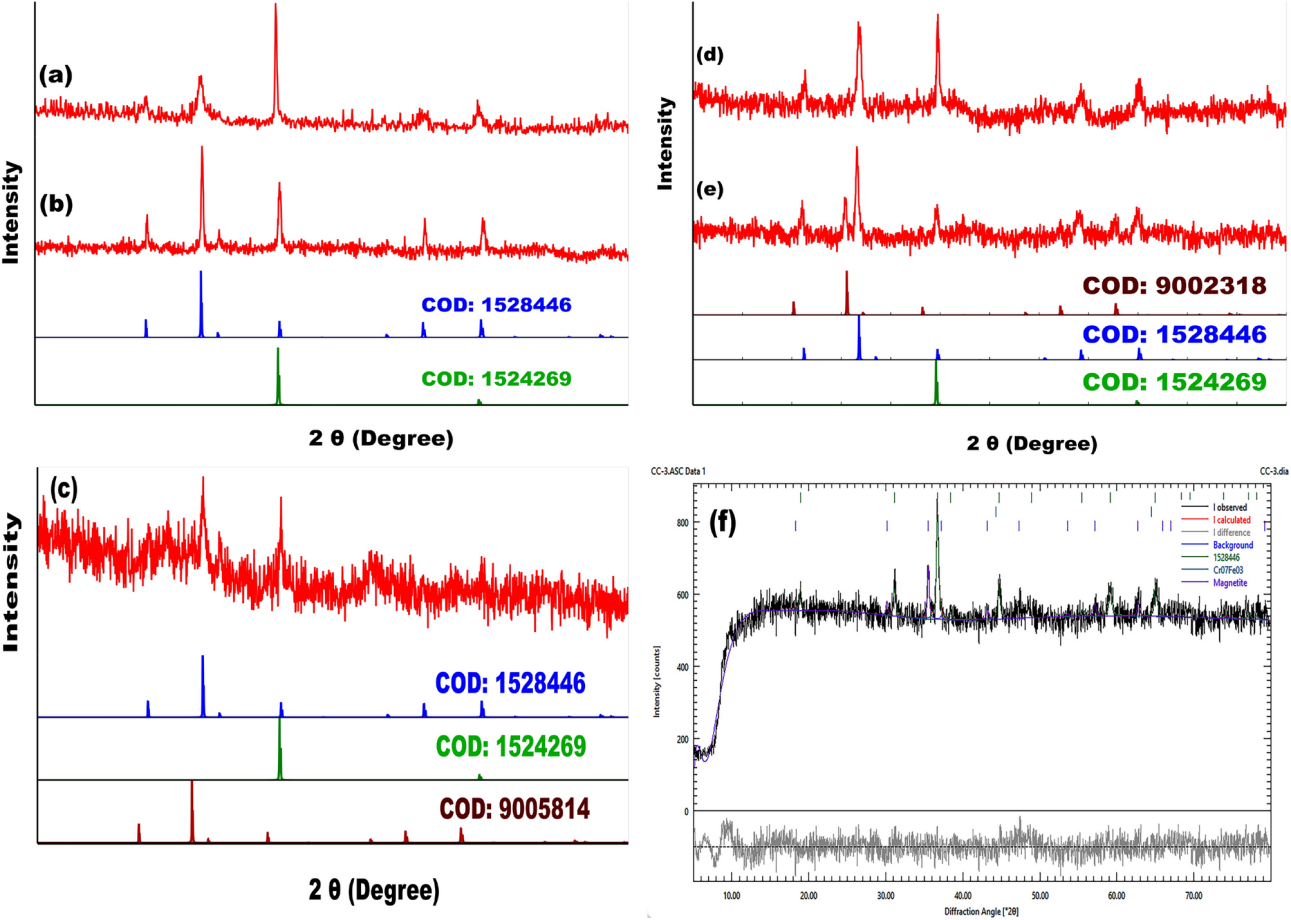
XRD spectra of (**a**) SiCo-1, (**b**) SiCo-2, (**c**) SiCoFe-1, (**d**) SiCoFe-2, and (**e**) SiCoFe-3, and (**f**) Rietveld analysis of SiCoFe-3.

Although all nanomaterials show well-defined sharp peaks indicating good crystallinity, the presence of noise is due to the presence of amorphous silicon dioxide, Fig. 3a–e. Profex was used to execute the Rietveld refinement of SiCoFe-3 (see Table 3), using the structural file obtained from COD (Crystallography Open Database), Fig. 3f^62^. The XRD patterns of noncompounds showed Co_2.74_O_4_ phase which is matched by reference code COD: 1528446. SiCoFe-1, SiCoFe-2 and SiCoFe-3 showed the phase Fe_3_O_4_ which is matched by reference code COD: 9002318 (SiCoFe-2 and SiCoFe-3) and COD: 9005814 (SiCoFe-1). The sample holder (Cr_7_Fe_3_: COD 1524269) was also identified^63^. This phase was integrated in the refinement. All nanomaterials showed diffraction peaks in the $18.90^{\circ }$–$19.00^{\circ }$, $31.11^{\circ }$–$31.27^{\circ }$, $36.65^{\circ }$–$36.84^{\circ }$, $38.34^{\circ }$–$38.54^{\circ }$, $44.57^{\circ }$–$44.8^{\circ }$, $49.08^{\circ }$–$49.08^{\circ }$, $55.34^{\circ }$–$55.65^{\circ }$, $59.02^{\circ }$–$59.35^{\circ }$, $64.86^{\circ }$–$65.23^{\circ }$, $68.23^{\circ }$–$68.62^{\circ }$, $69.33^{\circ }$–$69.73^{\circ }$, $73.68^{\circ }$–$74.11^{\circ }$, $76.87^{\circ }$–$77.33^{\circ }$, and $77.93^{\circ }$–$78.40^{\circ }$ regions which may be indexed to (1 1 1), (2 2 0), (3 1 1), (2 2 2), (4 0 0), (3 3 1), (4 2 2), (3 3 3), (4 4 0), (5 3 1), (4 4 2), (6 2 0), (5 3 3), and (6 2 2), respectively for Cubic symmetry group of Co_2.74_O_4_ with space group F d -3 m (227). The XRD spectra of SiCoFe-1, SiCoFe-2, and SiCoFe-3 showed more peaks in the $18.34^{\circ }$–$18.41^{\circ }$, $30.16^{\circ }$–$30.29^{\circ }$, $35.53^{\circ }$–$35.68^{\circ }$, $37.16^{\circ }$–$37.32^{\circ }$, 43.18$^{\circ }$–43.37$^{\circ }$, 47.28$^{\circ }$–47.49$^{\circ }$, 53.57$^{\circ }$–53.81$^{\circ }$, 57.11$^{\circ }$–57.37$^{\circ }$, 62.71$^{\circ }$–63.01$^{\circ }$, 65.94$^{\circ }$–66.25$^{\circ }$, 67.00$^{\circ }$–67.32$^{\circ }$, 71.15$^{\circ }$–71.50$^{\circ }$, 74.20$^{\circ }$-74.56$^{\circ }$, 75.20$^{\circ }$-75.57$^{\circ }$, and 79.18$^{\circ }$-79.58$^{\circ }$ regions which may be indexed to (1 1 1), (2 2 0), (3 1 1), (2 2 2), (4 0 0), (3 3 1), (4 2 2), (3 3 3), (4 4 0), (5 3 1), (4 4 2), (6 2 0), (5 3 3), (6 2 2), and (4 4 4), respectively for Cubic symmetry group of Fe3O4 with space group F d -3 m (227). The crystallite sizes of Co_2.74_O_4_ were 53.05, 35.38, 45.49, 53.05, and 31.84 nm in SiCo-1, SiCo-1, SiCoFe-1, SiCoFe-2 and SiCoFe-3, respectively, while that of Fe_3_O_4_ were 45.35, 39.66, and 31.73 in SiCoFe-1, SiCoFe-2 and SiCoFe-3, respectively. These Values were calculated using Scherrer’s formula using FWHM of the peaks with maximum intensities. The structural parameters of SiCo-1, SiCo-2, SiCoFe-1, SiCoFe-2, and SiCoFe-3 are listed in Table 4.

**Table 3:** Atomic and structural parameters.

Sample	Refinement parameters		Phase (COD)	Element	X	Y	Z	Occ.	Lattice parameter	Cell Volume (Å3)
SiCoFe-3	R_wp_	4.49	Co_2.74_O_4_ (1528446)	Co_1_	0.12500 (0.12500)	0.12500 (0.12500)	0.12500 (0.12500)	0.820 (0.820)	a= b= c= 8.10820 (8.09790), $\alpha$ = $\gamma$ = $\beta$= 90.00	533.06 (531.03)
				Co_2_	0.50000 (0.50000)	0.50000 (0.50000)	0.50000 (0.50000)	0.960 (0.960)		
	R_exp_	4.34								
				O	0.26130 (0.26130)	0.26130 (0.26130)	0.26130 (0.26130)	1.000 (1.000)		
	X^2^	1.07	Fe_3_O_4_ (9002318)	Fe_1_	0.12500 (0.12500)	0.12500 (0.12500)	0.12500 (0.12500)	1.000 (1.000)	a= b= c= 8.37150 (8.36850), $\alpha$ = $\gamma$ = $\beta$= 90.00	586.69 (586.06)
				Fe_2_	0.50000 (0.50000)	0.50000 (0.50000)	0.50000 (0.50000)	1.000 (1.000)		
	G_oF_	1.03								
				O	0.25500 (0.25500)	0.25500 (0.25500)	0.25500 (0.25500)	1.000 (1.000)

**Table 4:** Structural parameters.

Sample	2$\theta$	FWHM	Microstrain $\epsilon$ x 10^-3^	Specific surface area (m^2^ g^-1^)	Lorentz factor L_f_	Thomson polarization parameter I_P_	Lorentz polarization parameter L_P_
SiCo-1 (Co_2.74_O_4_)	36.65	0.16	2.06	20.05	2.66	0.82	17.52
SiCo-1 (Co_2.74_O_4_)	36.78	0.23	3.07	30.07	2.65	0.82	17.38
SiCoFe-1 (Co_2.74_O_4_)	36.78	0.18	2.39	23.39	2.65	0.82	17.38
SiCoFe-1 (Fe_3_O_4_)	35.68	0.18	2.47	25.21	2.80	0.83	18.58
SiCoFe-2 (Co_2.74_O_4_)	36.69	0.16	2.05	20.05	2.66	0.82	17.48
SiCoFe-2 (Fe_3_O_4_)	35.53	0.21	2.83	28.83	2.82	0.83	18.75
SiCoFe-3 (Co_2.74_O_4_)	36.73	0.26	3.42	33.41	2.65	0.82	17.43
SiCoFe-3 (Fe_3_O_4_)	35.54	0.26	3.54	36.03	2.82	0.83	18.74

### TEM, SEM and EDX

**Fig. 4:**
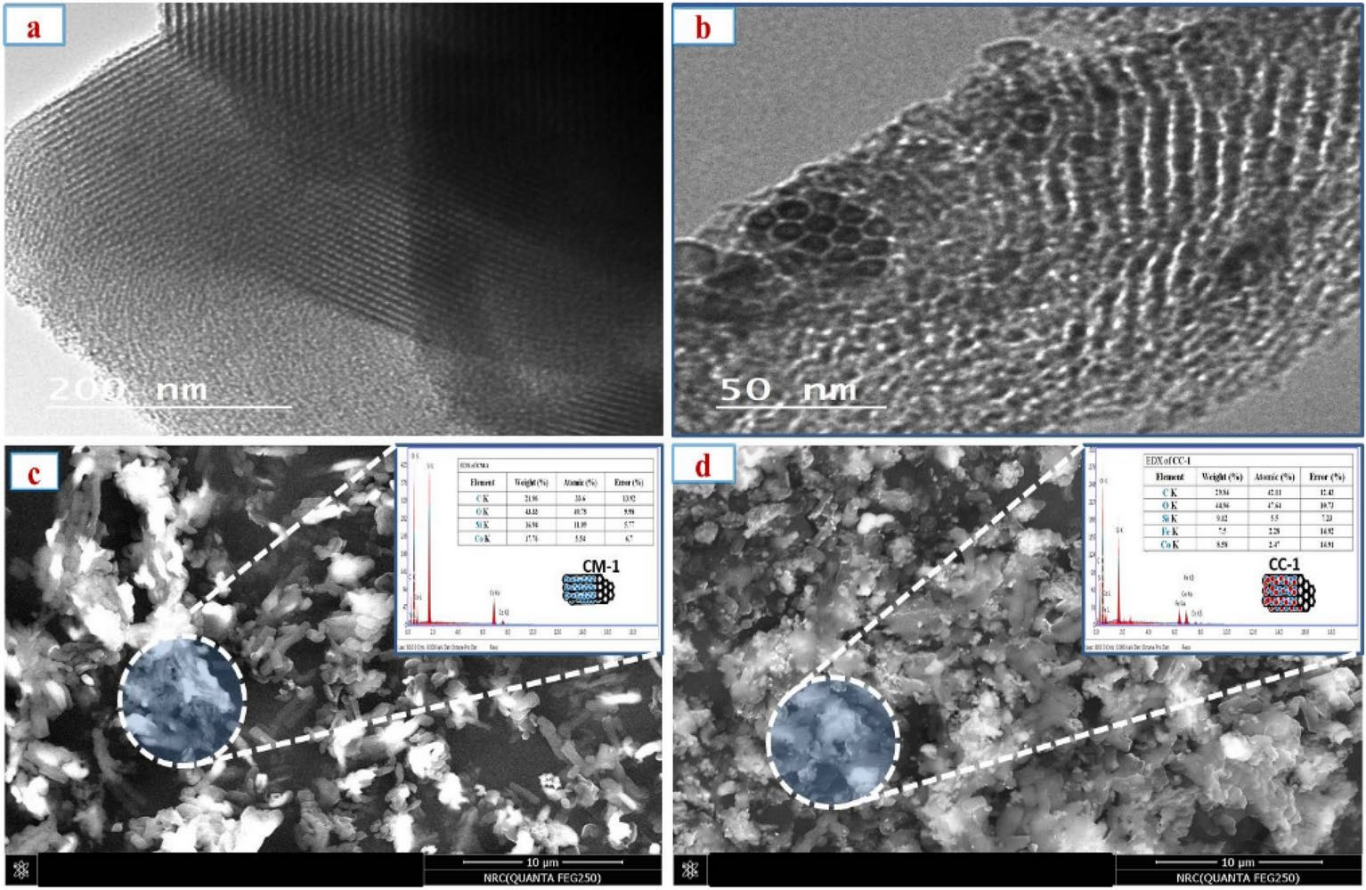
TEM (**a**) SiCo-1, (**b**) SiCoFe-3, SEM (**c**) SiCo-1, (**d**) SiCoFe-1.

TEM analysis was used to investagate the nanoparticles’ structure and get specific information about their distribution inside the SBA-15 Fig. 4a,b. Initially, it is worth noting that all Metal oxide@SBA-15 solids have characteristic and well-organized SBA-15 structures. These structures consist of cylindrical mesochannels with a restricted range of sizes. No significant clumps were found on the outer surface of the silica grains, which confirms that careful drying of the metal precursor/silica mixtures is beneficial for containing and stabilizing the CoO and Fe$_{2}$O$_{3}$ particles within the small pores of SBA-15. Additionally, this drying method helps prevent the movement of the oxide phase to the outer surface during the heating process. In addition, the oxide nanoparticles exhibit a homogeneous and evenly spread distribution within the porous host grains. They take the form of rod-shaped particles with a diameter of approximately 5–9 nm, which is consistent with the pore diameter of the SBA-15 host. This is illustrated in Fig. 4c,d. The particle shape described was previously documented for cobalt and manganess oxides nanowires produced inside SBA-15 mesopores using the “two solvent” technique^21,64^. The SEM images show the shape and surface appearance of Co-Fe@SBA-15. Figure 4c,d show the SEM of SiCo-1 and SiCoFe-1, respectively, showing differet morphological structure. Unlike TEM, which identifies the point where the beam enters the particle, the SiCoFe-1 SEM picture is shown in Fig. 4d. SiCoFe-1 seldom aggregates, suggesting that Co and Fe NPs are well dispersed throughout the mesoporous SBA-15 structure. The comparison between SEM of SiCo-1 and SiCoFe-1, indecate that SiCo-1 is less agrigated and have more uniformed particles shape and size. SiCo-1 show bacillary-shaped particles with two distinct contrast areas indecating the presence of two elements. SiCoFe-1 show irregular particles, relatively wide size distribution and highly porous surface with mashroom-lik texture. Additionally, the EDX analysis of SiCo-1 and SiCoFe-1 is shown in Fig. 4c,d. The signals for the EDX spectrum were gathered very quickly, and SiCoFe-1 displayed distinctive cobalt and iron absorption peaks at 0.550 keV, 0.750 keV, and 6.500 keV, 7 keV, confirming the presence of cobalt and iron nanoparticles^65,66^.

### $\gamma$-ray shielding properties

**Fig. 5:**
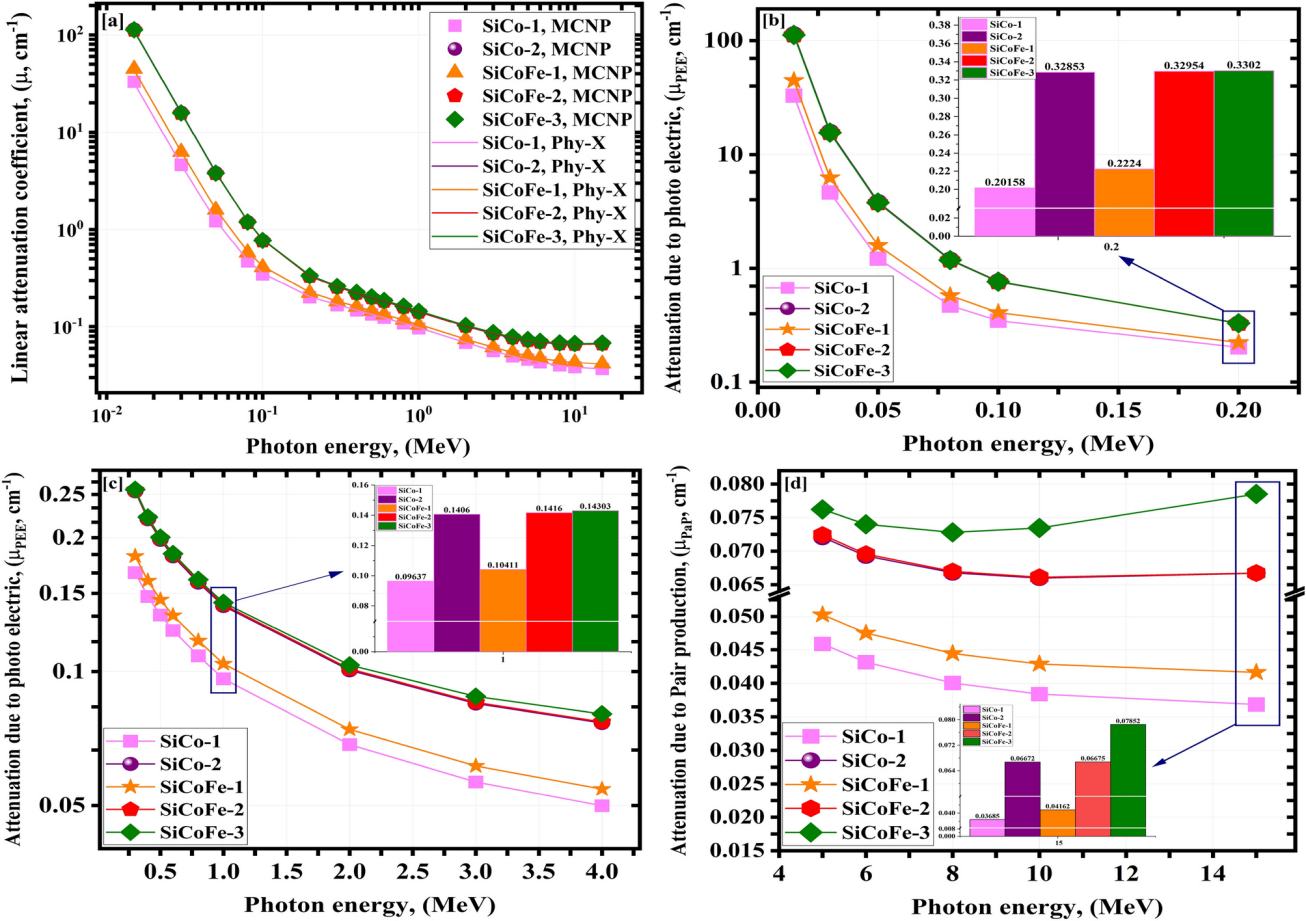
Influence of $\gamma$-ray energy on LA of (**a**) obtained from MCN and PhyX, (**b**) photo-electric, (**c**) compton scattering, and (**d**) pair production for the synthetic SiCoFe samples.

Figure 5 shows LA of the five synthetic SiCoFe samples. Figure 5a represents the LA using MCN and PhyX (E$_{\gamma }$ 0.015: 15 MeV). The LA values are in good agreement with the values calculated by PhyX ($\Omega _{max}$ = 3.569 %), as listed in Table 5. Increasing E$_{\gamma }$ values (0.015 - 15 MeV) decreased the simulated LA values of the synthetic samples (SiCo-1 (32.950: 0.037 cm$^{-1}$), SiCo-2 (113.673: 0.067 cm$^{-1}$), SiCoFe-1 (44.648: 0.042 cm$^{-1}$), SiCoFe-2 (112.113: 0.067 cm$^{-1}$), and SiCoFe-3 (112.129: 0.079 cm$^{-1}$)). This agree with results obtained for Ce/Dy oxides lead borate glasses (M1 (491.429: 0.271 cm$^{-1}$), M2 (458.103: 0.250 cm$^{-1}$), M3 (440.423: 0.244 cm$^{-1}$), M4 (419.991: 0.232 cm$^{-1}$)), and copper oxide/silver borosilicate glass (CuB-1 (226.02: 0.12 cm$^{-1}$), CuAgB-2 (227.88: 0.12 cm$^{-1}$), CuAgB-3 (229.62: 0.12 cm$^{-1}$), and CuAgB-4 (237.71: 0.13 cm$^{-1}$)) in the same energy ranges^67,68^.

Figure 5b showed that, Pee caused a tough decrease in the LA and cross-section ($\sigma$)values with the enrichment of E$_{\gamma }$ values. Increasing E$_{\gamma }$ values (0.015 - 15 MeV) causes a tough exponential decreasing tendency (SiCo-1 (32.950: 0.202 cm$^{-1}$), SiCo-2 (113.673: 0.329 cm$^{-1}$), SiCoFe-1 (44.648: 0.222 cm$^{-1}$), SiCoFe-2 (112.113: 0.330 cm$^{-1}$), and SiCoFe-3 (112.129: 0.330 cm$^{-1}$))

Figure 5c, indicate a exponential fall in LA values in the energy $\gamma$-photon interval from 0.300 MeV to 4.000 MeV, which is caused by the enrichment of E$_{\gamma }$ over 0.200 MeV. The exponential decline resulted from COM interacting with the changes in $\sigma$ caused by E. As the energy of a $\gamma$-photons highly increase leading to a greater velocity, its propensity of interacting with material’s atoms decreases and hence scattering increases. The enrichment in E$_{\gamma }$ values (0.300: 4.000 MeV) led to a decrease in $\sigma$, the quantity of interacting $\gamma$-electron and finaly LA values (SiCo-1 (0.167: 0.050 cm$^{-1}$), SiCo-2 (0.254: 0.077 cm$^{-1}$), SiCoFe-1(0.182: 0.054 cm$^{-1}$), SiCoFe-2 (0.256: 0.077 cm$^{-1}$), and SiCoFe-3 (0.256: 0.080 cm$^{-1}$)).

As shown in Fig. 5d, the interaction of PaP with $\sigma$ changes with E$_{\gamma }^{2}$ (5.000: 15.000 MeV) has caused a slight reduction in LA values (SiCo-1 (0.046: 0.037 cm$^{-1}$), SiCo-2 (0.072: 0.067 cm$^{-1}$), SiCoFe-1 (0.050: 0.042 cm$^{-1}$), SiCoFe-2 (0.072: 0.067 cm$^{-1}$), and SiCoFe-3 (0.076: 0.079 cm$^{-1}$)).

From the previous results, adding high concentrations of cobalt-oxide and hematite increases the prepared glasses’ densities and also, LA significantly. Due to the high concentration of cobalt and iron (55.95% and 16.74% mol.) and their high effective atomic numbers (27 and 26), SiCoFe-3 outperforms the other samples in terms of LA values.

**Table 5:** The LA, which was obtained using MCN and PhyX for the synthetic glasses.

Energy Mev	SiCo-1			SiCo2			SiCoFe1			SiCoFe2			SiCoFe3		
	Phy-x	MCNP5	%dif	Phy-x	MCNP5	%dif	Phy-x	MCNP5	%dif	Phy-x	MCNP5	%dif	Phy-x	MCNP5	%dif
0.015	31.8395	32.9497	3.3692	109.9569	113.6729	3.2690	43.2824	44.6478	3.0582	108.8092	112.1126	2.9465	109.0589	113.0955	3.5692
0.030	4.6441	4.6298	0.3092	15.9310	15.8660	0.4094	6.2826	6.2439	0.6203	15.7541	15.6397	0.7320	15.7820	15.7648	0.1092
0.050	1.2382	1.2183	1.6345	3.8841	3.8179	1.7348	1.6229	1.5919	1.9456	3.8449	3.7673	2.0573	3.8534	3.7989	1.4345
0.080	0.4838	0.4717	2.5548	1.2252	1.1935	2.6550	0.5942	0.5776	2.8658	1.2178	1.1826	2.9775	1.2233	1.1951	2.3548
0.100	0.3547	0.3470	2.2305	0.7861	0.7681	2.3414	0.4204	0.4100	2.5523	0.7838	0.7634	2.6640	0.7887	0.7729	2.0412
0.200	0.2047	0.2016	1.5507	0.3340	0.3285	1.6509	0.2265	0.2224	1.8617	0.3360	0.3295	1.9734	0.3399	0.3354	1.3507
0.300	0.1685	0.1669	0.9351	0.2569	0.2543	1.0353	0.1840	0.1818	1.2461	0.2591	0.2556	1.3578	0.2624	0.2605	0.7351
0.400	0.1484	0.1476	0.5482	0.2214	0.2200	0.6484	0.1615	0.1601	0.8593	0.2235	0.2214	0.9710	0.2265	0.2257	0.3482
0.500	0.1346	0.1341	0.3730	0.1989	0.1979	0.4732	0.1462	0.1452	0.6841	0.2008	0.1993	0.7958	0.2035	0.2032	0.1730
0.600	0.1240	0.1237	0.2743	0.1824	0.1817	0.3746	0.1346	0.1338	0.5854	0.1842	0.1830	0.6971	0.1867	0.1866	0.0743
0.800	0.1086	0.1085	0.0813	0.1589	0.1586	0.1815	0.1177	0.1172	0.3924	0.1605	0.1597	0.5041	0.1627	0.1629	0.1187
1.000	0.0974	0.0964	1.1127	0.1423	0.1406	1.2129	0.1056	0.1041	1.4238	0.1438	0.1416	1.5355	0.1457	0.1444	0.9127
2.000	0.0686	0.0686	0.0928	0.1009	0.1007	0.1930	0.0745	0.0742	0.4038	0.1019	0.1014	0.5155	0.1033	0.1034	0.1072
3.000	0.0565	0.0565	0.1446	0.0848	0.0848	0.0444	0.0615	0.0614	0.1664	0.0855	0.0853	0.2781	0.0867	0.0870	0.3446
4.000	0.0498	0.0499	0.2382	0.0766	0.0767	0.1380	0.0545	0.0545	0.0728	0.0772	0.0771	0.1845	0.0782	0.0786	0.4382
5.000	0.0457	0.0459	0.2804	0.0719	0.0721	0.1802	0.0503	0.0503	0.0307	0.0725	0.0724	0.1424	0.0734	0.0738	0.4804
6.000	0.0430	0.0431	0.2408	0.0692	0.0693	0.1405	0.0475	0.0475	0.0703	0.0697	0.0696	0.1820	0.0705	0.0708	0.4408
8.000	0.0399	0.0400	0.4211	0.0666	0.0668	0.3209	0.0444	0.0444	0.1100	0.0670	0.0670	0.0017	0.0677	0.0682	0.6211
10.000	0.0382	0.0384	0.4117	0.0658	0.0660	0.3114	0.0428	0.0429	0.1006	0.0661	0.0661	0.0111	0.0668	0.0673	0.6117
15.000	0.0367	0.0368	0.3860	0.0665	0.0667	0.2858	0.0416	0.0416	0.0750	0.0668	0.0667	0.0367	0.0674	0.0678	0.5860

**Table 6:** $\mu$ values of the synthetic SiCoFe samples and other published glasses.

Sample	LA, cm$^{-1}$		
	0.5	5	10
SiCo1	0.1341	0.0459	0.0384
SiCo2	0.1980	0.0721	0.0660
SiCoFe1	0.1452	0.0503	0.0429
SiCoFe2	0.1993	0.0724	0.0661
SiCoFe3	0.2003	0.0762	0.0735
RS-253-G18	0.2220	0.0736	0.0603
RS-360	0.4960	0.1379	0.1485
BSLMFCu0	0.2071	0.0670	0.0516
BSLMFCu10	0.2278	0.0741	0.0573

**Fig. 6:**
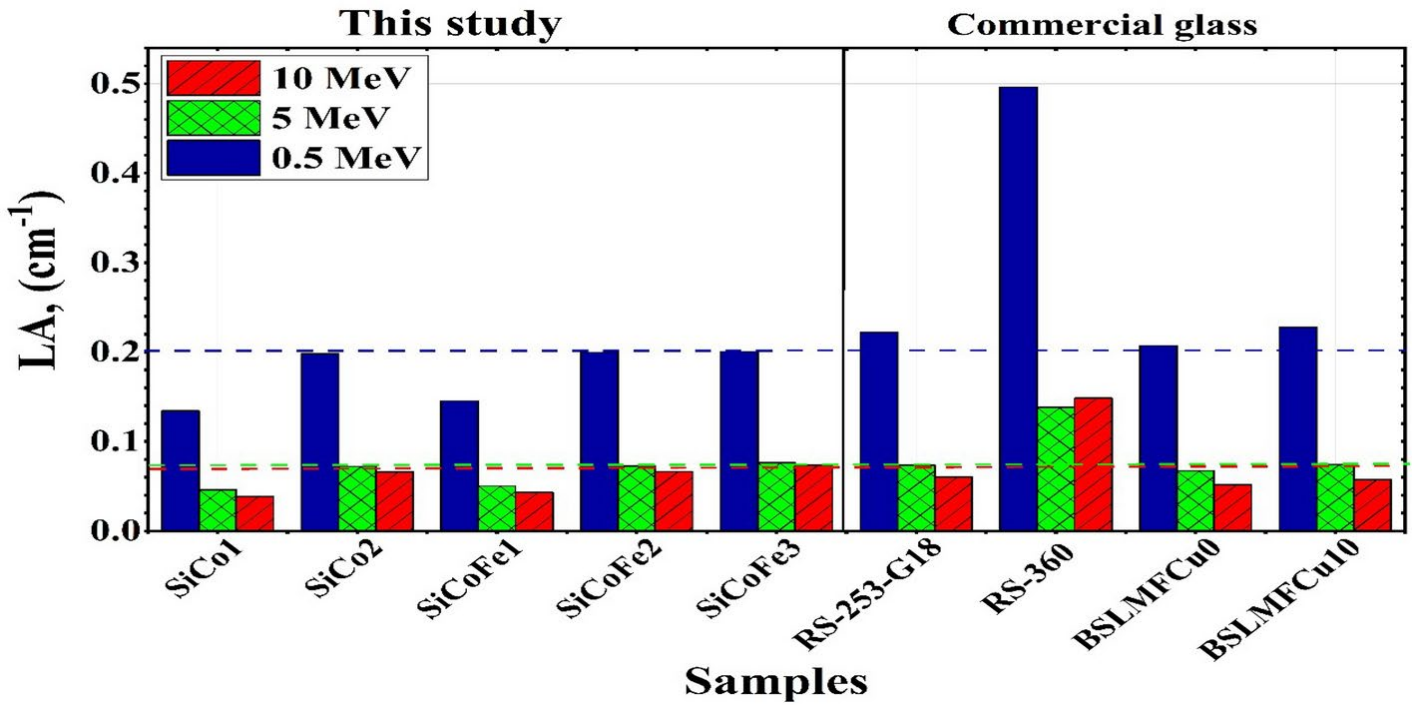
The LA (cm$^{-1}$) vs. $\gamma$-photon energy for the synthetic SiCoFe glasses compared with some reference glass samples.

Figure 6 and Table 6 compare the $\mu$ values of the synthetic SiCoFe samples and other published glasses (BSLMFCu0, BSLMFCu10, RS-253-G18, and RS-360) at chosen energies of 0.5, 5, and 10 MeV^27,29^ . At 0.5 MeV, the $\mu$ of the synthetic SiCoFe sample has lower values than those of RS-253-G18, BSLMFCu10, and RS-360 glasses. At 5, and 10 MeV, the $\mu$ of the synthetic SiCoFe3 sample has a higher value than other samples and is lower than the RS-360 sample.

**Fig. 7:**
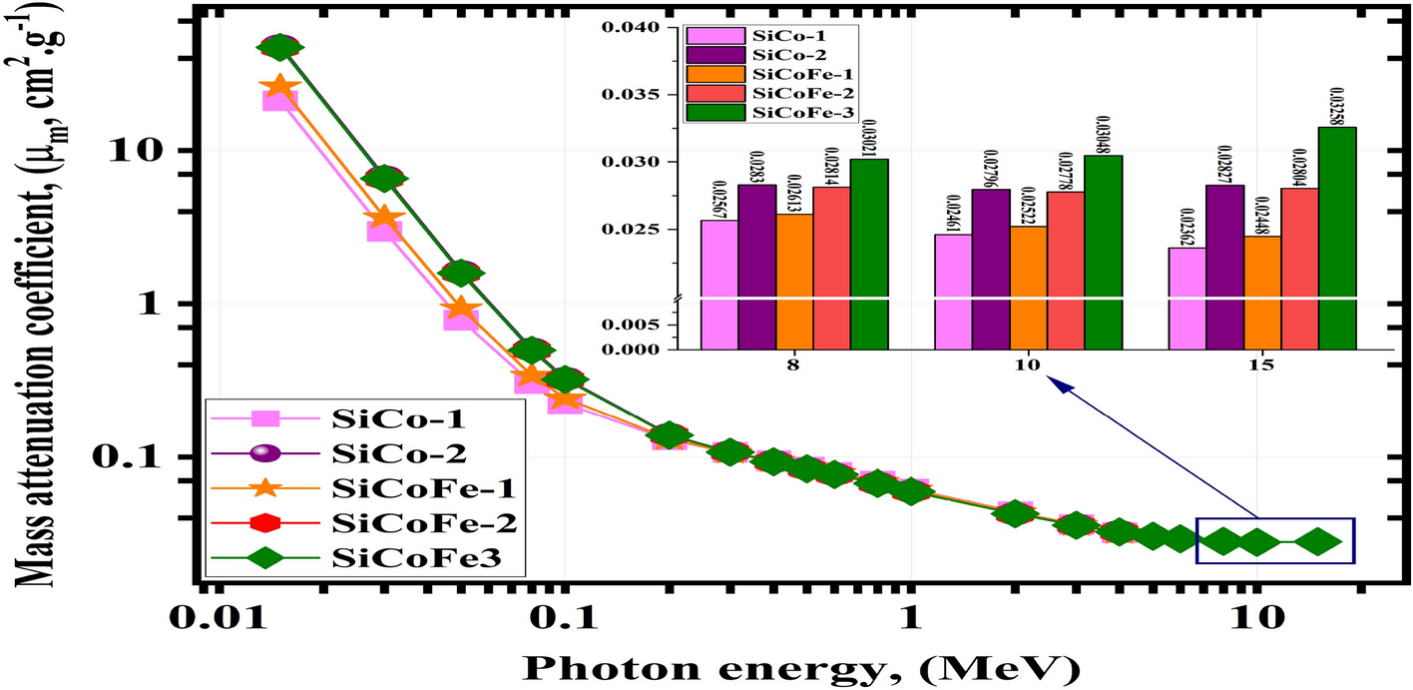
The mass attenuation (MA) obtained from MCN vs. the $\gamma$-photon energy for the synthetic SiCoFe glasses.

For the studied glasses of SiCoFe, the MA and LA showed a similar behavior, see Fig. 7.

**Fig. 8:**
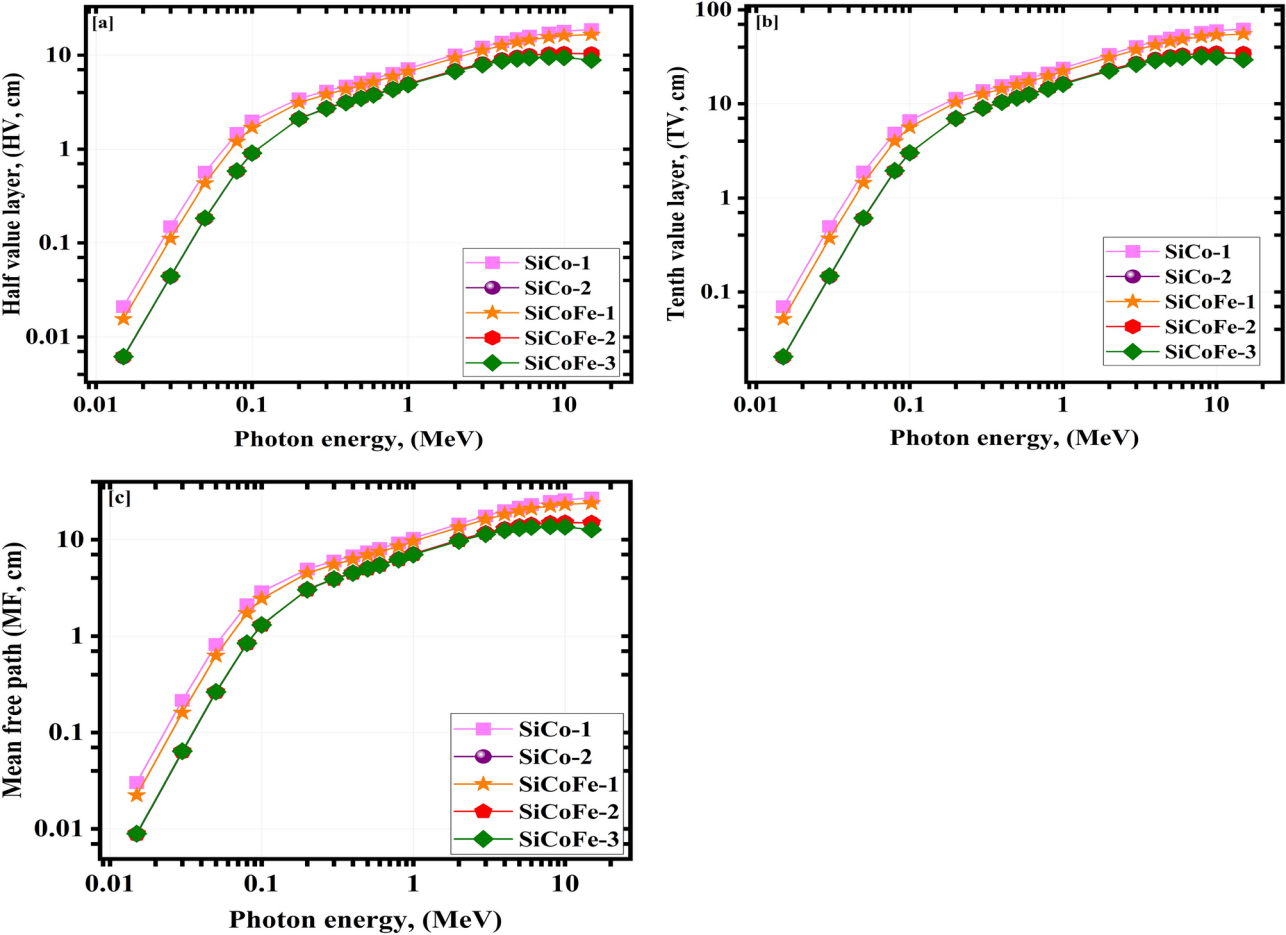
(**a**) The HV, (**b**) TV, and MF for the synthetic SiCoFe glasses vs. the $\gamma$-photon energy.

HV, TV and MF are used to indicate the efficiency of radiation protection, hence for a given E$_{\gamma }$, the lower their values, the stronger radiation shielding performance sine it travels through a narrower zone. Typically, the HV, TV, and MF all go up and down in tandem. Figure 8a–c presents the values of HV, TV, and MF computed by Eqs. (3), (4), and (5). The HV of investigated SiCoFe glasses increased as the LA values decreased.

The HV values (SiCo-1 (0.021: 18.810 cm), SiCo-2 (0.006: 10.388 cm), SiCoFe-1 (0.016: 16.655 cm), SiCoFe-2 (0.006: 10.385 cm), and SiCoFe-3 (0.006: 8.827 cm)) grew with raising the E$_{\gamma }$ values (0.015: 15 MeV) as seen in Fig. 8a.

The variations of TV with $\gamma$-photon energy are illustrated in Fig. 8b. The TV values range from 0.070 to 62.486 cm for SiCo-1, from 0.020 to 34.509 cm for SiCo-2, from 0.052 to 55.327 cm for SiCoFe-1, from 0.021 to 34.498 cm for SiCoFe-2, and from 0.021 to 29.323 cm for SiCoFe-3 sample. Figure 8c represents the MF of the examined glasses as it varies with energy. The MF values range from 0.030 to 27.137 cm, from 0.009 to 14.987 cm, from 0.022 to 24.028 cm, from 0.009 to 14.982 cm, and from 0.009 to 12.735 cm for the synthetic glasses SiCo-1, SiCo-2, SiCoFe-1, SiCoFe-2, and SiCoFe-3, respectively. From the obtained results, the HV, TV, and MF were found to be dependent on the Nano hematite and cobalt oxide content, which indicated that the HV, TV, and MF values reached the lowest values for the SiCoFe-3 sample. In addition, the nanocomposites with hematite and/or cobalt nanoparticles have better $\gamma$ attenuation capabilities within the selected $\gamma$-photon energy range. It also showed the role these nanofillers perform in attenuating $\gamma$-rays.

**Fig. 9:**
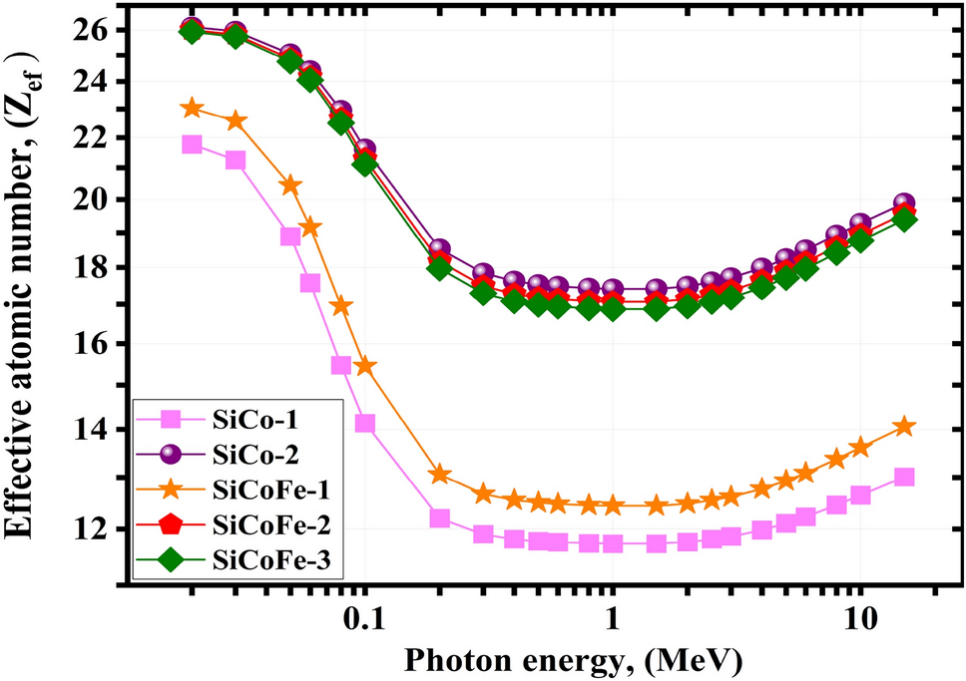
The Z$_{ef}$ for the synthetic SiCoFe glasses vs. $\gamma$-photon energy.

A composite’s Z$_{ef}$ is useful in several fields, including physics, and modern technology. A material with high Z$_{ef}$ value may block high-energy radiation, mainly via the COM effect and PEE^69,70^ and hence have enhanced radiation interaction. For the energy spectrum of interest, the range of Z$_{ef}$ of the synthetic SiCoFe glasses varied from 21.410–13.010, 26.120–19.890, 23.020–14.060, 26.000–19.570, and 25.93–19.570 for the synthetic glasses SiCo-1, SiCo-2, SiCoFe-1, SiCoFe-2, and SiCoFe-3, respectively, see Fig. 9. Although current system has a lower Z$_{ef}$ than some systems including the silver doped glass CuB-1 (74.89–51.29), CuAgB-2 (76.22–52.16), CuAgB-3 (76.43–52.29) and CuAgB-4 (76.67–52.45), their is a high cost differance between them. The composition of this glass also contains many other oxides including copper, zinc, lithium, and boron oxides, indicating a higher doping power.^67^

The synthetic glasses; SiCo-2, SiCoFe-2, and SiCoFe-3 have the highest Z$_{ef}$ values among the E$_{\gamma }$ range from 0.015: 15 MeV due to the high doping with the cobalt oxides.

**Fig. 10:**
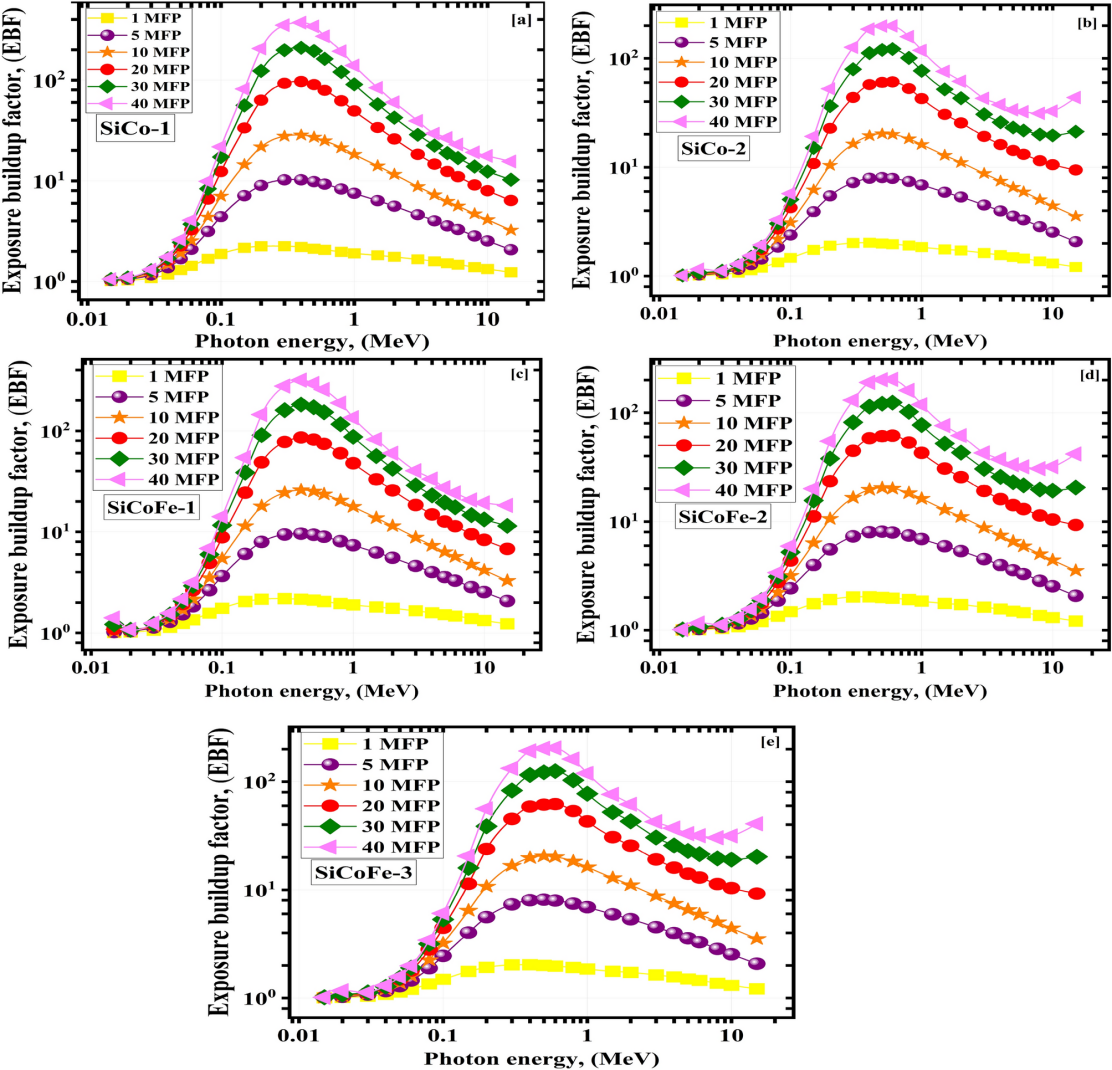
The exposure buildup factor (EBF) vs. $\gamma$-photon energy for the synthetic SiCoFe glasses (**a**) SiCo-1, (**b**) SiCo-2, (**c**) SiCoFe-1, (**d**) SiCoFe-2, and (**e**) SiCoFe-3.

**Fig. 11:**
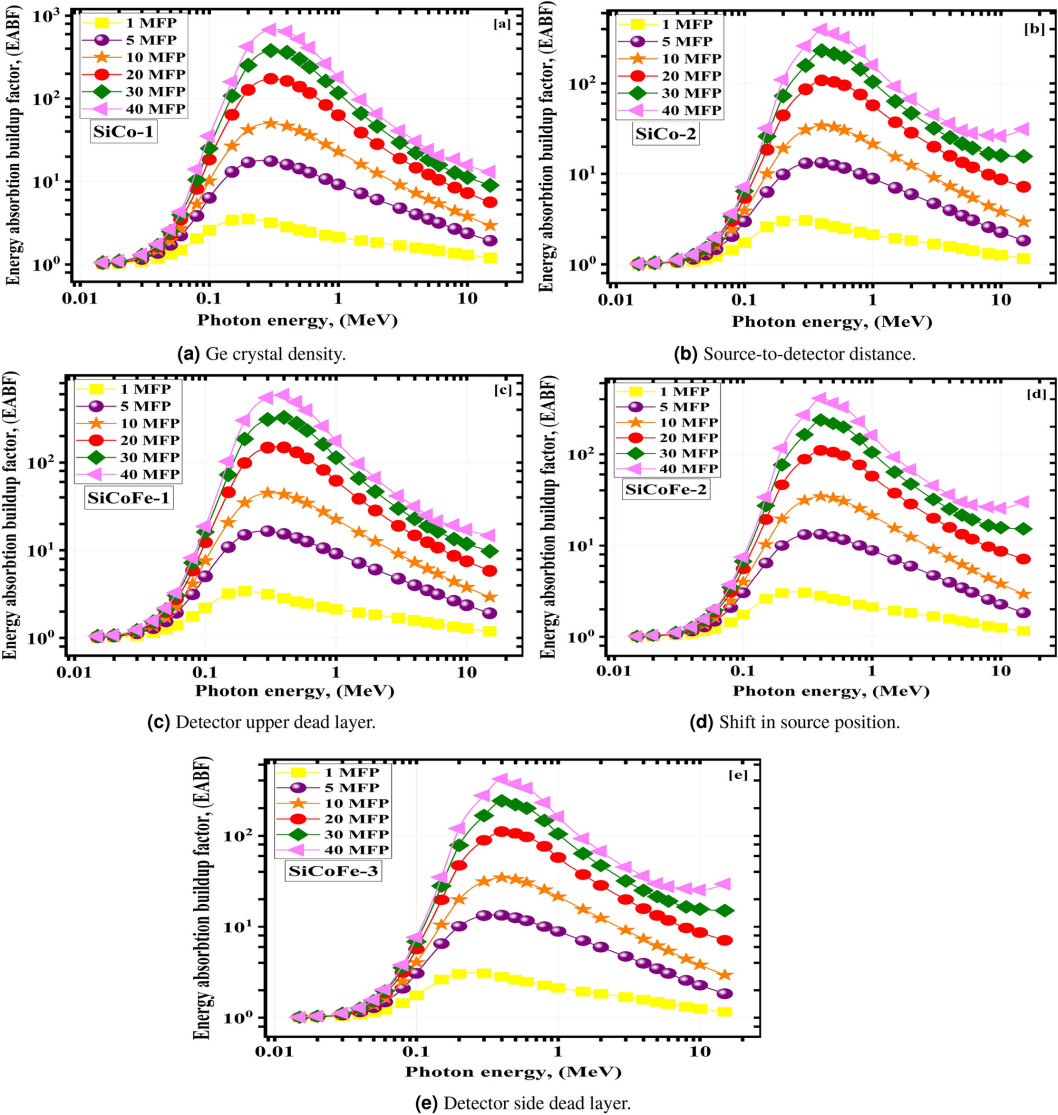
The energy absorption buildup factor (EABF) vs. $\gamma$-photon energy for the synthetic SiCoFe glasses (**a**) SiCo-1, (**b**) SiCo-2, (**c**) SiCoFe-1, (**d**) SiCoFe-2, and (**e**) SiCoFe-3.

The energy absorption exposure buildup factors (EABF and EBF) values change with $\gamma$-photon energy for the substances being studied at 1, 5, 10, 15, 20, 30, and 40 MF. They are shown in Figs. 10 and 11. $\gamma$-photon energy, sample composition, and penetration depth all play a role in determining the upper limits of the BUFs. At larger depths, multiple scatterings occur^71^. The 40 MFs penetration depth yielded the greatest BUF readings, while the 1 MFs depth yielded the lowest. The BUF values increase with $\gamma$-photon energy up to a maximum, then decrease with further increases in $\gamma$-photon energy. Many $\gamma$-photons have been absorbed, and the BUFs are the lowest because the Pee dominates interactions at low energies^33,72,73^. The largest BUF values are seen in the intermediate $\gamma$-photon energy range because the prevalent COM scatters the E$_{\gamma }$ but cannot destroy it. The $\gamma$-photons were again absorbed in the higher energy area, where PaP is the main interaction.

The highest LA values obtained with SiCo2 (83.2% Co$_{2.74}$O$_{4}$, 0% Fe$_{2}$O$_{3}$, total doping 83.2%) < SiCoFe-2 (71.7% Co$_{2.74}$O$_{4}$, 13.8% Fe$_{2}$O$_{3}$, total doping 85.5%) < SiCoFe-3 (63.6% Co$_{2.74}$O$_{4}$, 23.9% Fe$_{2}$O$_{3}$, total doping 87.5%). The order agrees with doping power. The variation of Co$_{2.74}$O$_{4}$: Fe$_{2}$O$_{3}$ ratios did not show a significant impact on LA values because the very close effective atomic numbers (27 and 26) of cobalt and iron, respectively

## Conclusion

This research introduces a significant advancement in radiation protection technology through the development of novel binary and ternary nano cobalt and iron oxides nanocomposites based on mesoporous silica (SBA-15).

Our systematic approach employed template-assisted sol-gel synthesis to create hexagonally ordered silica nanorods as the foundation for these shielding materials. The resulting binary (SiCo-1, SiCo-2) and ternary (SiCoFe-1, SiCoFe-2, SiCoFe-3) nanocomposites were characterization using XRD, XPS, TEM, and SEM analyses.

The XRD patterns investigation revealed the successful formation of Co$_{2.74}$O$_{4}$ (COD: 1528446) and Fe$_{3}$O$_{4}$ (COD: 9002318 (SiCoFe-2 and SiCoFe-3) and 9005814 (SiCoFe-1)) phases. The Rietveld refinement parameters (R$_{w}$=4.49, R$_{ex}$=4.34, x$^{2}$=1.07, G$_{of}$=1.03) confirming excellent structural quality. XPS survey indicated the purity of the samples. XPS indicated the presence of Fe as Fe$_{2}$O$_{3}$ and the presence of cobalt as Co$^{2+}$ and Co$^{3+}$ with the later ranging from 9.19% to 74.70%.

Most significantly, our gamma radiation shielding assessments using Monte Carlo simulations and Phy-X software revealed that the concentration and combination of metal oxides dramatically influence shielding performance. The linear attenuation coefficients demonstrated a clear concentration-dependent relationship, with higher cobalt and iron oxide content yielding superior shielding capabilities. The $\gamma$-shielding results confirmed that:

- The LA order is: SiCo-1 (0.1341, total doping 0% ) < SiCo-2 (0.1980, total doping 83.2%) >SiCoFe-1 (0.1452, total doping 47.4% ) < SiCoFe-2 (0.1993, total doping 85.5% ) < SiCoFe-3 (0.2003, total doping 87.5% ).
- The HV of the synthesized SiCoFe-3 sample has the lowest HV, TV, and MF.
- Within the investigated energy range of Z$_{ef}$ within the range: 21.410–13.010, 26.120–19.890, 23.020–14.060, 26.000–19.570, and 25.93–19.570 for the synthetic glasses SiCo-1, SiCo-2, SiCoFe-1, SiCoFe-2, and SiCoFe-3, respectively.
- The SiCoFe-3 composition emerged as the premier shielding material among all synthesized variants, exhibiting the highest linear attenuation coefficient and the lowest half-value layer.
- Comparing the oxides ratio and doping power of composites with the highest LA values we obtain:
  - SiCo2 (83.2% Co$_{2.74}$O$_{4}$, 0% Fe$_{2}$O$_{3}$, total doping 83.2%) < SiCoFe-2 (71.7% Co$_{2.74}$O$_{4}$, 13.8% Fe$_{2}$O$_{3}$, total doping 85.5%) < SiCoFe-3 (63.6% Co$_{2.74}$O$_{4}$, 23.9% Fe$_{2}$O$_{3}$, total doping 87.5%)
  - Composites are ordered according to the increase in doping power (as stated before).
  - Varying The Co$_{2.74}$O$_{4}$: Fe$_{2}$O$_{3}$ ratios did not show a high impact on LA values.

Depending on current results, the highest doping of Co$_{2.74}$O$_{4}$ ration (83.2%) may be invastigated with other co-doping oxides other than Fe$_{2}$O$_{3}$.

## Data Availability

Data is provided within the manuscript.
